# Supplementary material for: Comprehensive Annotation and Functional Exploration of MicroRNAs in Lettuce
Source: Front Plant Sci. 2021 Dec 24;12:781836. doi: 10.3389/fpls.2021.781836 (PMC8739914; doi:10.3389/fpls.2021.781836)
Supplement: Supplementary file 3 [file Data_Sheet_9.ZIP › GSM2230751.plot/Lsa-miR396a_Lsat_1_v5_gn_8_161820.1_1050_TPlot.pdf]

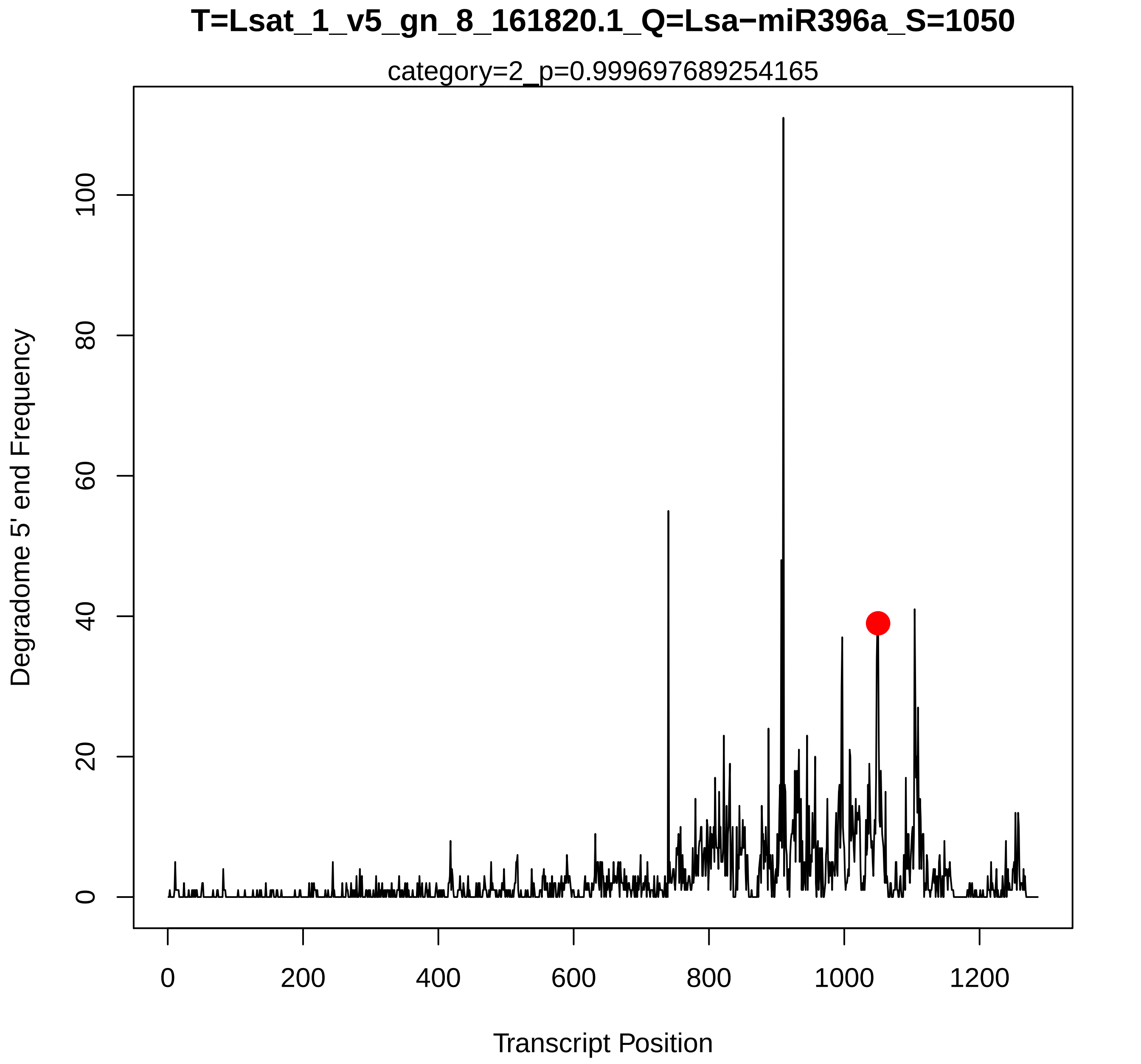

T=Lsat_1_v5_gn_8_161820.1_Q=Lsa−miR396a_S=1050
category=2_p=0.999697689254165
Degradome 5' end Frequency
0
20
40
60
80
100
0
200
400
600
800
1000
1200
Transcript Position
